# Heterogeneous autoregressive model with structural break using nearest neighbor truncation volatility estimators for DAX

**DOI:** 10.1186/s40064-016-3465-x

**Published:** 2016-11-06

**Authors:** Wen Cheong Chin, Min Cherng Lee, Grace Lee Ching Yap

**Affiliations:** 1 Faculty of Management, SIG Quantitative Economics and Finance, Multimedia University, 63100 Cyberjaya, Selangor Malaysia; 2 Lee Kong Chian Faculty of Engineering and Science, Universiti Tunku Abdul Rahman, 43300 Kajang, Selangor Malaysia; 3 Faculty of Engineering, University of Nottingham (Malaysia Campus), 43500 Semenyih, Selangor Malaysia

**Keywords:** Structural break, Nearest neighbor truncation estimator, Heterogeneous autoregressive model

## Abstract

High frequency financial data modelling has become one of the important research areas in the field of financial econometrics. However, the possible structural break in volatile financial time series often trigger inconsistency issue in volatility estimation. In this study, we propose a structural break heavy-tailed heterogeneous autoregressive (HAR) volatility econometric model with the enhancement of jump-robust estimators. The breakpoints in the volatility are captured by dummy variables after the detection by Bai–Perron sequential multi breakpoints procedure. In order to further deal with possible abrupt jump in the volatility, the jump-robust volatility estimators are composed by using the nearest neighbor truncation approach, namely the minimum and median realized volatility. Under the structural break improvements in both the models and volatility estimators, the empirical findings show that the modified HAR model provides the best performing in-sample and out-of-sample forecast evaluations as compared with the standard HAR models. Accurate volatility forecasts have direct influential to the application of risk management and investment portfolio analysis.

## Background

With recent enhancement of information technology, the high frequency financial data are more accessible to academician and investors. The availability of high frequency data in financial time series has great contribution to the accuracy of volatility estimations especially in the applications of finance (Cervelló-Royo et al. 2015; Dionne et al. 2015; Liu and Tse 2015; Louzis et al. 2014). One of the important literatures is written by Andersen and Bollerslev (1998) who have introduced the high frequency realized volatility (RV) by cumulating the sum of products of squared returns within a day. However, the RV estimation becomes inconsistent (Barndorff-Nielsen and Shephard 2004) for integrated volatility under the presence of abrupt jumps (structural breaks). There is ample empirical evidence on this phenomenon in financial markets (Duonga and Swanson 2015; Ewing and Malik 2016; Barunika et al. 2016; Dendramis et al. 2015). The structural break may cause by voluminous drastic feedbacks from market participants due to new inflow market information. The sudden shifts mostly related to large positive/negative market return shocks which include the leverage effect (Charles and Darne 2014), risk premium (Dendramis et al. 2014) and even financial crisis (Klose 2014). Ignoring the presence of structural breaks may cause serious misleading statistical results such as incorrect descriptive statistics, erroneous hypothesis inferences, unreliable forecasts, just to mention a few.

There are two approaches to deal with the structural break in financial time series. *First*, is to use robust-jump volatility estimators and *secondly* is to embrace the structural break feature in the econometric models. *Firstly*, one may select volatility estimators which are robust to abrupt jumps. Barndorff-Nielsen and Shephard (2004) introduced the multipower variation (MPV) volatility with the cumulative sum of products of most adjacent absolute returns. The MPV is robust to jumps because the product of consecutive returns has a smaller impact of jump after the averaging processes. However, the MPV is still sensitive and bias to the presence of very small returns. Recently, Andersen et al. (2012) have introduced two jump-robust estimators using the nearest neighbor truncation (NNT) approach to battle this issue. The first volatility estimator, minimum realized volatility (minRV) is constructed by scaling the square of the minimum of two consecutive absolute returns. With the presence of jump during an interval, the minRV will eliminate it and compute based on the adjacent diffusive returns. Again, minRV is also sensitive to very small returns and leads to efficiency issue. Consequently, to improve the robustness to jump, the median realized volatility (medRV) uses the median operator to square the median of three consecutive absolute returns. In other words, the minimum and median operators intended to eliminate the extreme noise of volatility. In short, one may use the high frequency MPV and NNT estimators to deal with structural break in the volatility representative. For the *second* remedy, one may use the econometric models that directly deal with structural breaks. These include jump stochastic volatility model (Dendramis et al. 2015), HAR-jump (Andersen et al. 2007), HAR-regime smooth transition (McAleer and Medeiros 2008) and Markov-switching ARFIMAX (Martens et al. 2009).

In this study, we include both the aforementioned methods in the standard heterogeneous autoregressive (HAR) proposed by Corsi et al. (2008). This model assumes that the financial markets consist of heterogeneous market participants with *short* (noise traders and speculators), *medium* (portfolio managers and hedge fund managers) and *long* (long term portfolio managers and pension fund managers) trading horizon investments. It is in accordance with the concept of heterogeneous market hypothesis (HMH) recommended by Muller et al. (1993) and Dacorogna et al. (2001) where the informationally market efficiency is explained under the assumption of heterogeneous market participants. One of the interesting statistical properties of HAR is the long memory volatility which created by the cascades of different investment horizon activities. Another interesting phenomenon of finance which can be explained by the HMH is on how the market liquidity is formed. Under the HAR framework, the time-varying market liquidity can be captured according to the dominant investment horizons. The trading among heterogeneity market participants with different views on the same security’s value is the key to form a liquid market. In normal market conditions, the short investment horizons investors focus on technical analysis whereas long investment horizons investors judge from the fundamental information for a same security. For instance, a negative inflowing news may be an indicator of selling for short horizon investors, but might be a buying opportunity for long horizon investor, and vice versa. If there are sufficient buying and selling among these investors, the financial market can be considered under an equilibrium or stable condition. However during economic crisis (e.g. Subprime mortgage crisis), the structure of equilibrium is disturbed where long horizon investors are either quit or become short horizon investors. Great selling activities by short horizon investors (due to exogenous event) has caused drastic drop in prices. These unusual plunges have negative impact for the economy prospects and long horizon investors has doubt the validity of their view of the economic fundamentals. Consequently, they might quit or to join on overwhelmingly short horizon market dynamics. In short, the long memory property in HAR is diminished when a financial turmoil hits the market. In addition, the partial removal of long horizon investors has also caused the market become less liquid as the structure of heterogeneity is no longer exist.

In this study, we propose to combine both the robust-jump volatility estimator and structural break heterogeneous autoregressive (HAR) models to battle the structural break in stock market volatility modelling. The selected volatility estimators are based on the nearest neighbor truncation (NNT) approach namely the median (medRV) and minimum (minRV) realized volatility. For structural break HAR model, we firstly identify the multi-break points using the Bai and Perron (2003) approach and then embrace them in the standard HAR using dummy variables. In addition, the HAR model is equipped with other stylized fact features such as volatility clustering and fat-tailed property. It is worth noting that the proposed method in this study is somewhat different from the well-known approaches by Andersen et al. (2007), Corsi and Renò (2012) and Patton and Sheppard (2011) where the HAR volatility components are decomposed into continuous sample path variation and discontinuous break variation. As a comparison with the standard realized volatility, the modified HAR model provides better in-sample as well as out-of-sample forecast evaluations. This study aims to add the empirical literature of high frequency volatility analysis by using modified HAR models and robust-jump volatility estimators. The remaining of this study is organized as follows: “Methods” section provides the description of modified HAR model specification, estimation, diagnostic and forecast evaluations; “Result and discussion” section discusses the empirical data and results and finally, “Summary and conclusion” section concludes the findings of this study.

## Methods

### High frequency volatility formulations

Integrated volatility estimation based on high frequency data is commonly used to measure the latent volatility. Let’s consider a stochastic volatility process for logarithmic prices of an asset, *dp*(*t*) = *μ*(*t*)*dt* + *σ*(*t*)*dW*(*t*), where $\mu \left( t \right),\; \sigma \left( t \right)$ and *W*(*t*) are the drift, volatility and standard Brownian motion respectively. The *μ*(*t*) and *σ*(*t*) may be time-varying but are assumed to be independent of *dW*(*t*). The changes of logarithmic price is defined as the continuously compounded intraday returns of day *t* with sampling frequency *N* as $r_{t,j} = 100 \left( {\ln P_{t,j} - \ln P_{t,j - 1} } \right),$ a with *j* = 1, …, *N* − 1. In another form, $p_{t} = p_{0} + \int_{0}^{t} {\mu \left( t \right)dt} + \int_{0}^{t} {\sigma \left( t \right)dW\left( t \right)} .$ The quadratic variation process for a sequence of partitions when *N* approaches infinity is equivalent to the integrated variance $\lim\nolimits_{N \to \infty } \sum\nolimits_{i=1}^{N} {\left( {P_{{t_{i} }} - P_{{t_{i - 1} }} } \right)^{2} } = \int_{0}^{t} {\sigma^{2} \left( t \right)dt.}$ Under this condition, the integrated variance can be consistently estimated by the Realized Volatility (Andersen and Bollerslev 1998), *RV* = ∑ _*j*=1_^*N*^ *r*_*t*,*j*_^2^. For jump-robust estimators, Andersen et al. (2012) proposed minimum (minRV) and median (medRV) operators using the nearest neighbour truncation (NTT) approach to estimate the integrated volatility:

1 $$minRV_{t,N} = \frac{\pi }{\pi - 2}\left( {\frac{N}{N - 1}} \right)\mathop \sum \limits_{j = 1}^{N - 1} \left[ {{ \hbox{min} }\left( {\left| {r_{t,j} } \right|,\left| {r_{t,j + 1} } \right|} \right)} \right]^{2}$$

2 $$medRV_{t,N}^{{}} = \frac{\pi }{6 - 4\sqrt 3 + \pi }\left( {\frac{N}{N - 2}} \right)\mathop \sum \limits_{j = 2}^{N - 1} \left[ {med\left( {\left| {r_{t,j - 1} } \right|,\left| {r_{t,j} } \right|,\left| {r_{t,j + 1} } \right|} \right)} \right]^{2}$$

For i.i.d block of returns, the scaling factors ensure that each of the estimators provides an unbiased estimate of the underlying latent volatility. Since the block size is considerably small (minRV with blocks of two returns and MedRV with blocks of three returns), therefore they are still asymptotically valid. However, if the block size increases to a wider interval, the iid assumption become harder to maintain. The minimum realized volatility (minRV) eliminates a jump for a given block of two consecutive returns and compute based on the adjacent diffusive returns whereas the median realized volatility (medRV) uses the median operator to square the median of three consecutive absolute returns. Under the presence jump, these endogenous adaptive truncation volatility estimators have better theoretical efficiency properties and better finite-sample robustness than RV. In this specific study, the standard 5-min interval data are used to avoid microstructure noise issue. The impact of market microstructure noise can be further analyzed using the higher frequency data such as 1- or 2-min interval.

### The heavy-tailed HAR–GARCH model with structural break

In order to identify the breakpoints in the long-run level of volatility representations, we have selected the Bai–Perron sequential procedures (Bai and Perron 2003) in the full empirical sample. Assume that there are *m*-breaks with respective location *k*_*j*_, where $j = 1, 2, \ldots , m,$ the detection is based on the ordinary least squared standard HAR model:

3 $$\begin{aligned} { \ln }(RV_{t}^{d}) & = \mu_{0} + \mu_{1} { \ln }\left({RV_{t - 1}^{d}} \right) + \mu_{2} { \ln }\left({RV_{t - 2}^{d}} \right) + \mu_{3} { \ln }\left({RV_{t - 1}^{w}} \right) + \mu_{4} { \ln }\left({RV_{t - 1}^{m}} \right) + \epsilon_{t} \\ { \ln }\left({minRV_{t}^{d}} \right) & = \mu_{0} + \mu_{1} { \ln }\left({minRV_{t - 1}^{d}} \right) + \mu_{2} { \ln }\left({minRV_{t - 2}^{d}} \right) + \mu_{3} { \ln }\left({minRV_{t - 1}^{w}} \right) + \mu_{4} { \ln }\left({minRV_{t - 1}^{m}} \right) + \epsilon_{t} \\ { \ln }\left({medRV_{t}^{d}} \right) & = \mu_{0} + \mu_{1} { \ln }\left({medRV_{t - 1}^{d}} \right) + \mu_{2} { \ln }\left({medRV_{t - 1}^{w}} \right) + \mu_{3} { \ln }\left({medRV_{t - 1}^{m}} \right) + \epsilon_{t} \\ \end{aligned}$$

where $\epsilon_{t}$ is the error. The detection procedure begins with the full sample under the parameter consistency test. When the test rejects the null hypothesis of consistency, the first breakpoint is determined and the full sample is divided into two samples. After that repeat the consistency test in each of the sub-samples as a test of the alternative of *m* + 1 = 2 versus the null hypothesis of *m* = 1 breaks. Terminate the procedures until all of the sub-samples do not reject the null hypothesis. When the number (*m*) and location (*k*_*j*_) of breaks have been identified, a dummy variable will included in both the intercept (level) and slope (heterogeneous components). The additional impact of the breaks can be measured by the estimated *μ* with its respective component. In this specific study, we begin with the maximum number of breakpoints as five. However, we only found one breakpoint (refer to Table 2) with significant impact to the volatility level and slope parameters in this study. After the sequential breakpoints have been identified, the heavy-tailed HAR–GARCH(1,1) model under the structural break can be written as:

4 $$\begin{aligned} { \ln }(RV_{t}^{d} )& = \theta_{C1} + \theta_{break,C2} *DUM_{k} + \theta_{d1,day} { \ln }(RV_{t - 1}^{day} ) + \theta_{d2,day} { \ln }(RV_{t - 2}^{day} ) \\ & \quad +\,\frac{1}{5}\theta_{w1,week} \left( {\mathop \sum \limits_{j = 1}^{5} lnRV_{t - j}^{day} } \right) + \frac{1}{22}\theta_{m1,month} \left( {ln\mathop \sum \limits_{j = 1}^{22} RV_{t - j}^{day} } \right) + \theta_{break - d1,day} { \ln }(RV_{t - 1}^{day} ) \\ & \quad *\,DUM_{k} + \theta_{break - d2,day} { \ln }(RV_{t - 2}^{day} )*DUM_{k} + \frac{1}{5}\theta_{break - w1,week} \left( {\mathop \sum \limits_{j = 1}^{5} lnRV_{t - j}^{day} } \right)*DUM_{k} \\ & \quad +\,\frac{1}{22}\theta_{break - m1,month} \left( {ln\mathop \sum \limits_{j = 1}^{22} RV_{t - j}^{day} } \right)*DUM_{k} + a_{i,t} \\ \end{aligned}$$

5 $$\begin{aligned} { \ln }(minRV_{t}^{d} ) &= \theta_{C1} + \theta_{break,C2} *DUM_{k} + \theta_{d1,day} { \ln }(minRV_{t - 1}^{day} ) + \theta_{d2,day} { \ln }(minRV_{t - 2}^{day} ) \\ & \quad +\,\frac{1}{5}\theta_{w1,week} \left( {\mathop \sum \limits_{j = 1}^{5} lnminRV_{t - j}^{day} } \right) + \frac{1}{22}\theta_{m1,month} \left( {ln\mathop \sum \limits_{j = 1}^{22} minRV_{t - j}^{day} } \right) \\ & \quad +\,\theta_{break - d1,day} { \ln }(minRV_{t - 1}^{day} )*DUM_{k} + \theta_{break - d2,day} { \ln }(minRV_{t - 2}^{day} )*DUM_{k} \\ & \quad +\,\frac{1}{5}\theta_{break - w1,week} \left( {\mathop \sum \limits_{j = 1}^{5} lnminRV_{t - j}^{day} } \right)*DUM_{k} + \frac{1}{22}\theta_{break - m1,month} \left( {ln\mathop \sum \limits_{j = 1}^{22} minRV_{t - j}^{day} } \right) \\ & \quad *\,DUM_{k} + a_{i,t} \\ \end{aligned}$$

6 $$\begin{aligned} { \ln }(medRV_{t}^{d} )& = \theta_{C1} + \theta_{break,C2} *DUM_{k} + \theta_{d1,day} { \ln }(medRV_{t - 1}^{day} ) + \frac{1}{5}\theta_{w1,week} \left( {\mathop \sum \limits_{j = 1}^{5} lnmedRV_{t - j}^{day} } \right) \\ & \quad +\,\frac{1}{22}\theta_{m1,month} \left( {ln\mathop \sum \limits_{j = 1}^{22} medRV_{t - j}^{day} } \right) + \theta_{break - d1,day} { \ln }(medRV_{t - 1}^{day} )*DUM_{k} \\ & \quad +\,\theta_{break - d2,day} { \ln }(medRV_{t - 2}^{day} )*DUM_{k} + \frac{1}{5}\theta_{break - w1,week} \left( {\mathop \sum \limits_{j = 1}^{5} lnmedRV_{t - j}^{day} } \right)*DUM_{k} \\ & \quad +\,\frac{1}{22}\theta_{break - m1,month} \left( {ln\mathop \sum \limits_{j = 1}^{22} medRV_{t - j}^{day} } \right)*DUM_{k} + a_{i,t} \\ \end{aligned}$$

with the GARCH specifications:

7 $$\begin{aligned} a_{i,t} & = \sigma_{i,t} \varepsilon_{i,t} ,\quad \varepsilon_{i,t} \;\sim\;GED \\ \sigma_{i,t}^{2} & = \alpha_{i,0} + \alpha_{i,1} a_{i,t - 1}^{2} + \beta_{i,} \sigma_{i,t - 1}^{2} \\ \end{aligned}$$

where *i* = 1, 2, 3 denotes the volatility representation for RV, minRV and medRV. The dummy variable is defined as *DUM*_*k*_ = 1 if the observation falls on breakpoint *k* and onwards whereas 0 otherwise. Based on the HAR specification, the current volatility is cascaded by previous daily, weekly and monthly volatilities and the GARCH component, *σ*_*i*,*t*_^2^ can be interpreted as the volatility of RV (Corsi et al. 2008). Due to the non-normality issue commonly observed in financial time series, we assume that the error *a*_*t*_ follows a generalized error distribution (Nelson 1991) under the maximum likelihood estimation with the density function for both the models as follows:

8 $$f\left( {z;v} \right) = \frac{{ve^{{\left( { - \frac{1}{2}\left| {\frac{{z_{t} }}{\lambda }} \right|^{v} } \right)}} }}{{\lambda 2^{{\left( {\frac{1 + v}{v}} \right)}} {{\Gamma }}\left( {\frac{1}{v}} \right)}}.$$

where Γ[·] is the gamma function and $\lambda = \left( {\frac{{2^{ - 2/v} {\Gamma [}v^{ - 1} \text{]}}}{{{\Gamma [3}v^{ - 1} \text{]}}}} \right)^{0.5}$ with *v* < 2 for heavier tail as compared to normal distribution *v* = 2. Using the Ox-G@RCH, the estimations are conducted using the maximum likelihood by the Broyden, Fletcher, Goldfarb and Shanno (BFGS) unconstrained optimization method. Overall, the vector parameters to be estimated for HAR are $\widehat{{\Theta}}\left( {\varvec{\theta},\varvec{\alpha},v} \right)$ where $\varvec{\theta}= \left( {\theta_{0} ,\theta_{1} ,\theta_{2} , \theta_{d} , \theta_{w} ,\theta_{m} ,\theta_{jump} } \right)$ and $\varvec{\alpha}= \left( {\alpha_{0} , \alpha_{1} ,\beta_{1} } \right)$ respectively.

For model diagnostic, the Ljung–Box serial correlations are used to examine the standardized and squared standardized residuals under the null hypothesis of uncorrelated series. Next, the model selections are based on the Akaike information criterion $\left( {AIC = - 2\frac{{L_{T} }}{T} + 2\frac{k}{T}} \right)$, Schwarz information criterion $\left( {SIC = - 2\frac{{L_{T} }}{T} + 2\frac{{{ \ln }\,\left( k \right)}}{T}} \right)$ and Hannan–Quinn information criterion $\left( {IC = - 2\frac{{L_{T} }}{T} + 2\frac{{k{ \ln }\,\left( {\ln \left( k \right)} \right)}}{T}} \right)$ which are evaluated from the adjusted (penalty function due to additional number estimated parameters) average log likelihood function (*L*_*T*_). After the in-sample forecast evaluation, the out-of-sample forecast evaluations is based on I-1-day ahead forecast where $h = 1, 2, \ldots ,H$ with *H* fixed to 230. In order to evaluate the best out-of-sample forecast, we have selected root mean squared error (RMSE), mean absolute error (MAE), mean absolute percentage error (MAPE) and Theil inequality coefficient (TIC) to indicate the power of predictability. In this study, we follow the robustness definition by Patton (2011) where the model ranking should be consistent no matter what types of proxies are being used as actual values in the forecast evaluations. In order to obtain a fair and objective forecast evaluation, we alternately use the RV, minRV and medRV as the proxy of actual volatility in all the three measurements. A simple scoring scheme is used to accumulate their scores and then rank them accordingly.

## Result and discussion

For empirical study, we have selected the DAX index using the Bloomberg database started from 1st February 2008 until 27th February 2015 with a total of 1799 observations. For high frequency data, we have selected the 5-min data to reduce the microstructure effect. The daily realized volatility accumulated 105 5-min data with approximately 190,000 5-min data for 1799 trading days. This includes the out-of-sample forecast evaluations data from 3rd February 2014 to 27th February 2015. It is noted that we have included the subprime mortgage crisis period started from early year of 2008 to ensure that the empirical data is highly volatile with possible jumps in the series.

**Fig. 1:**
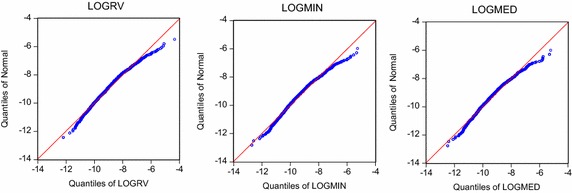
Quantile–quantile plots for various RVs versus normal distribution

Table 1 and Fig. 1 show that all the series are statistically deviated from normal distribution. Therefore, a non-Gaussian distributed innovation should be considered in the model specification. For break point identification, initially we pre-specified five (5) breakpoints. After the detections, only two of the coefficients of dummy variables are significantly different from zero in the HAR models. Table 2 indicates the sequential F-statistics and their respective locations for each volatility representations.

**Table 1:** Descriptive statistics for various logarithmic RVs

Statistic	LOG(RV)	LOG(minRV)	LOG(medRV)
Mean	−8.980892	−9.420352	−9.403562
Median	−9.037955	−9.493426	−9.467515
SD	1.005797	0.990786	0.980879
Skewness	0.494192	0.507808	0.512527
Kurtosis	3.724067	3.712591	3.692619
Jarque–Bera	112.5255*	115.3804*	114.7205*

$${\text{Jarque}}{{-}}{\text{Bera}}\;{\text{statistic}} = \frac{T}{6}\left( {skewness + \frac{{Kurtosis - 3^{2} }}{4}} \right)$$

* 5 % level of significance

**Table 2:** Multi-breakpoint detections

Breakpoint	LogRV		LogminRV and logmedRV	
	F-statistic	Date	F-statistic	Date
*k*_1_	21.64299	13/June/2012	22.32691	3/Aug/2012

Critical values (Bai and Perron 2003) for *k*_1_ is 18.23 for RV and 16.19 for minRV and medRV at 0.05 level

### Estimation results

Tables 3, 4 and 5 report the estimation results for standard HAR–GARCH(1,1)-Normal, structural break HAR–GARCH(1,1)-Normal and structural break HAR–GARCH(1,1)-GED for RV, minRV and medRV respectively. These various models allow us to verify the advantages of including the structural break in the HAR and GARCH specifications. As indicated in Table 5, all the tail indexes for generalized error distribution (GED) are less than 2 which suggested that the innovations of volatility are fat-tailed.

**Table 3:** Estimation for standard HAR–GARCH(1,1)-NORMAL

Estimation	LogRV	LogminRV	LogmedRV
*θ*_*C*1_	−0.535111* (0.154328)	−0.463228* (0.142400)	−0.463000* (0.141844)
Heterogeneous component: *θ*_*d*1,*day*_	0.217124* (0.033015)	0.354625* (0.032161)	0.378463* (0.031435)
*θ*_*d*2,*day*_	0.128490* (0.033799)	0.088655* (0.036500)	0.059026 (0.035999)
*θ*_*w*1,*week*_	0.382793* (0.066941)	0.303845* (0.063905)	0.327452* (0.061893)
*θ*_*m*1,*month*_	0.211781* (0.043782)	0.204804* (0.040240)	0.186657* (0.039168)
GARCH component			
*α*_0_	0.010513* (0.003401)	0.037335* (0.013389)	0.010907* (0.004508)
ARCH effect, *α*_1_	0.044551* (0.009438)	0.075417* (0.017158)	0.047100* (0.010372)
GARCH effect, *β*_1_	0.923950* (0.015984)	0.782428* (0.062491)	0.908679* (0.025107)
Selection			
AIC	1.679927	1.484523	1.407996
SIC	1.707946	1.512527	1.436000
HIC	1.690358	1.494948	1.418421
Diagnose			
Q(10) for standardized *a*_*t*_	8.5638	7.3310	10.773
Q(10) for standardized *a*_*t*_^2^	7.183274	4.991463	5.390689

*, ** indicate 5 and 10 % level of significance respectively

**Table 4:** Estimation for heavy-tailed jump-robust HAR–GARCH(1,1)-NORMAL

Estimation	LogRV	LogminRV	LogmedRV
*θ*_*C*1_	−0.573789* (0.197060)	−0.572602* (0.175907)	−0.621407* (0.176719)
Heterogeneous component: *θ*_*d*1,*day*_	0.238119* (0.040082)	0.397943* (0.037278)	0.434135* (0.037851)
*θ*_*d*2,*day*_	0.149237* (0.041986)	0.075528** (0.042429)	
*θ*_*w*1,*week*_	0.392877* (0.078684)	0.302916* (0.071121)	0.347932* (0.055810)
*θ*_*m*1,*month*_	0.153743* (0.047953)	0.161430* (0.043576)	0.149333* (0.042079)
Break effect for			
*θ*_*break*, *C*1_	−1.488235* (0.536629)	−1.249370** (0.642890)	−1.175957* (0.606153)
*θ*_*break*−*d*1,*day*_	−0.087813 (0.068330)	−0.189498* (0.070081)	−0.195154** (0.066462)
*θ*_*break*−*d*2,*day*_	−0.077450 (0.070632)	0.035078 (0.082032)	
*θ*_*break*−*w*1,*week*_	−0.151890 (0.147371)	−0.018500 (0.164841)	0.053065 (0.119166)
*θ*_*break*−*m*1,*month*_	0.170241 (0.111817)	0.058175 (0.119320)	0.035186 (0.109871)
GARCH component:			
*α*_0_	0.010233* (0.003360)	0.020017* (0.008815)	0.010039* (0.004323)
ARCH effect, *α*_1_	0.040633* (0.009244)	0.048335* (0.013916)	0.043843* (0.010526)
GARCH effect, *β*_1_	0.928107* (0.015973)	0.874404* (0.043618)	0.915048* (0.024605)
Selection			
AIC	1.674161	1.478626	1.404343
SIC	1.719692	1.524157	1.442849
HIC	1.691111	1.495576	1.418677
Diagnostic			
Q(10) for standardized *a*_*t*_	7.9452	7.4558	14.238
Q(10) for standardized *a*_*t*_^2^	7.5748	6.2532	4.5178

*, ** indicate 5 and 10 % level of significance respectively

**Table 5:** Estimation for heavy-tailed jump-robust HAR–GARCH(1,1)-GED

Estimation	LogRV	LogminRV	LogmedRV
*θ*_*C*1_	−0.680556* (0.182853)	−0.593806* (0.169789)	−0.644560* (0.167535)
Heterogeneous component: *θ*_*d*1,*day*_	0.243567* (0.037212)	0.389279* (0.035970)	0.430507* (0.036053)
*θ*_*d*2,*day*_	0.146830* (0.039307)	0.070872** (0.040519)	
*θ*_*w*1,*week*_	0.381336* (0.074549)	0.310885* (0.068611)	0.347257* (0.053792)
*θ*_*m*1,*month*_	0.150149* (0.045796)	0.164232* (0.042325)	0.150838* (0.040687)
Break effect for			
*θ*_*break*,*C*1_	−1.228954* (0.565831)	−1.219960** (0.646179)	−1.109135** (0.606410)
*θ*_*break*−*d*1,*day*_	−0.068277 (0.066060)	−0.168897* (0.070700)	−0.182183* (0.067408)
*θ*_*break*−*d*2,*day*_	−0.059649 (0.068689)	0.065739 (0.079042)	
*θ*_*break*−*w*1,*week*_	−0.129702 (0.143583)	−0.059511 (0.157710)	0.061224 (0.116876)
*θ*_*break*−*m*1,*month*_	0.142415 (0.109048)	0.051767 (0.117073)	0.022046 (0.108647)
GARCH component			
*α*_0_	0.010233* (0.004372)	0.019657** (0.010186)	0.010080** (0.005338)
ARCH effect, *α*_1_	0.038397* (0.011729)	0.049301* (0.016547)	0.043678* (0.013165)
GARCH effect, *β*_1_	0.930122* (0.020874)	0.874745* (0.050479)	0.914798* (0.030635)
Tail index, λ	1.517782* (0.079217)	1.654018* (0.082734)	1.604856* (0.082409)
Selection			
AIC	1.656853	1.470345	1.392122
SIC	1.705886	1.519378	1.434128
HIC	1.675107	1.488599	1.407759
Diagnose			
Q(10) for standardized *a*_*t*_	8.2971	7.7389	14.462
Q(10) for standardized *a*_*t*_^2^	8.1479	6.4542	4.6093

*, ** indicate 5 and 10 % level of significance respectively

For HAR specification, the results shows that the heterogeneous autoregressive components (*θ*_*day*_, *θ*_*week*_ and *θ*_*month*_) for daily, weekly and monthly volatilities are all significantly different from zero at 5 % level of significance. Thus, this findings support heterogeneous market hypothesis (HMH) where the markets are constructed by heterogeneous market participants with different time horizon of investments. The past weekly volatility contributes strongest impact to the current daily volatility, follows by daily and monthly. For the structural break impact, all the models indicated significant level-break effect at 5 % level for the long run volatility. For instance, the long-run volatility (*θ*_*C*1_ = − 0.593806) for the structural break HAR–GARCH(1,1)-GED model (Table 5) obtained an additional impact (*θ*_*jump*,*C*1_ = −1.219960) under the presence of break. Besides the long-run volatility level, we also assume that the break is going to influence the heterogeneous components as well. In Tables 4 and 5, the empirical results show that only the minRV and medRV models for lag one daily heterogeneous components are affected by the presence of structural break. These empirical outcomes are acceptable since the financial markets often react (selling or buying activities) by the highly speculated market information (e.g. financial crisis, monetary policy changes, etc.) within a day. However, the market news after a week or a month normally have smaller impact to the market movements.

As a comparison, the structural break HAR–GARCH-GED model outperformed the rest of the models based on the three information criterion with the lowest results. Among the two NTT estimators, medRV performs better than its counterpart, the minRV. For diagnostic part, all the models failed to reject the Ljung–Box serial correlations for standardized innovations. As a summary, the HAR–GARCH(1,1)-GED is the most preferable model compared to others in the estimation. However, there is no guarantee this result will persist in the out-of-sample forecast evaluations due to other factors (Hong et al. 2004).

### Forecast evaluations

**Fig. 2:**
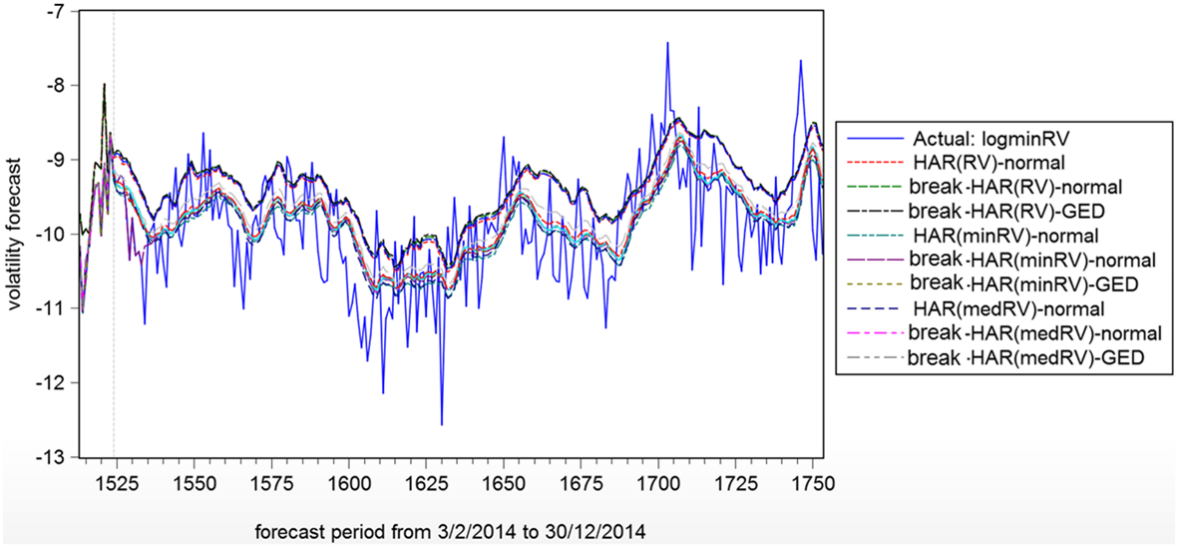
Dynamic forecast comparison for nine models

The out-of-sample consists of 230 one-ahead forecasts with the latent volatility is represented alternately by logRV, logminRV and logmedRV. This is to avoid the biasness issue of using only one actual volatility representations. Using the dynamic forecast approach, the estimated parameters will be used for the next one-day-ahead forecast. Table 6 and Fig. 2 reported the forecast evaluations for RMSE, MAE, MAPE and TIC for all the models.

**Table 6:** Forecast evaluations

	RMSE	MAE	MAPE	TIC
Actual: logRV				
HAR(RV)-normal	0.651281*	0.524890	5.562033	0.034283*
Break-HAR(RV)-normal	0.652070	0.521887*	5.509533	0.034394
​Break-HAR(RV)-GED	0.653979	0.522205	5.505789*	0.034520
HAR(minRV)-normal	0.764127	0.603088	6.624436	0.039294
​Break-HAR(minRV)-normal	0.731901	0.578977	6.343932	0.037724
​Break-HAR(minRV)-GED	0.729189	0.574511	6.292221	0.037625
HAR(medRV)-normal	0.746771	0.589072	6.460210	0.038443
​Break-HAR(medRV)-normal	0.718214	0.565700	6.187558	0.037089
​Break-HAR(medRV)-GED	0.714892	0.563258	6.158450	0.036932
Actual: logminRV				
HAR(RV)-normal	0.789156	0.644290	6.339814	0.040637
Jump-HAR(RV)-normal	0.809646	0.662120	6.504324	0.041774
​Break-HAR(RV)-GED	0.818384	0.670025	6.576982	0.042256
HAR(minRV)-normal	0.638405	0.506469	5.150216	0.032131
​Break-HAR(minRV)-normal	0.630824	0.492197*	4.980560*	0.031821
​Break-HAR(minRV)-GED	0.640285	0.508674	5.139286	0.032333
HAR(medRV)-normal	0.630286*	0.503166	5.103593	0.031756*
​Break-HAR(medRV)-normal	0.637293	0.508452	5.128620	0.032208
​Break-HAR(medRV)-GED	0.638447	0.509171	5.132373	0.032278
Actual: logmedRV				
HAR(RV)-normal	0.765429	0.623274	6.144211	0.039449
​Break-HAR(RV)-normal	0.786059	0.641008	6.307899	0.040592
​Break-HAR(RV)-GED	0.794619	0.648410	6.375548	0.041064
HAR(minRV)-normal	0.619110	0.494591	5.047823	0.031186
​Break-HAR(minRV)-normal	0.609756*	0.485405*	4.929134*	0.030784*
​Break-HAR(minRV)-GED	0.619592	0.496536	5.033198	0.031315
HAR(medRV)-normal	0.611312	0.488187	4.968397	0.030826
​Break-HAR(medRV)-normal	0.616746	0.493640	4.994146	0.031196
​Break-HAR(medRV)-GED	0.617718	0.494580	5.000038	0.031256

The best perform measurements are indicated by *

In general, the forecast evaluations can be examined in two aspects. First the type of actual volatility used in the forecast evaluations and second, the type of models based on the volatility representations, RV, minRV and medRV. For the *first scenario*, the forecast performances are in favor on the type of actual volatility used. For instance, when the logRV is used as the actual volatility, all the three models under logRV representation perform the best with mixture of normal and GED models. On the other hand, logminRV and logmedRV perform almost the same with the largest percentage error 17 % as compared to the best logRV models. When the actual forecasts shift to logminRV and logmedRV, the logminRV models show the best forecast evaluations, follow by logmedRV models and lastly the logRV models. It is worth to note that the logmedRV models only indicate 1–2 % of error as compared to logminRV whereas the logRV models show approximately 35 % error from the same actual forecasts using logminRV. The larger deviation of model logRV as compared to the other two NTT models may contribute from the nature of the noisiness which does not smoothen by the minimum and median operators. For the *second scenario*, the Jump-HAR(minRV) models with the normality assumption seem to perform better as compared to the GED assumption. However, it is worth noting that the performances for logminRV and logmedRV are very close with the deviation of 1–2 % of deviation from the best perform model.

## Summary and conclusion

This study combines two approaches to deal with structural breaks in the high frequency volatility modelling. Firstly, the structural break component is included in the HAR model and then secondly, using the jump-robust nearest neighbor truncation volatility estimators. Using these approaches, the proposed modified HAR model in general performs better than its standard form in both the in-sample and out-of-sample forecast evaluations. It is also worth noting that the forecast performances are also influence by the selected actual volatility in the forecast evaluations. In summary, this study provides valuable information to risk management and investment portfolio analysis where some of the finance applications such as value-at-risk can be determined directly from the volatility forecast results.

## References

[CR1] Andersen TG, Bollerslev T (1998). Answering the skeptics: yes, standard volatility models do provide accurate forecasts. Int Econ Rev.

[CR2] Andersen TG, Bollerslev T, Diebold FX (2007). Roughing it up: including jump components in the measurement, modeling and forecasting of return volatility. Rev Econ Stat.

[CR3] Andersen TG, Dobrev D, Schaumburg E (2012). Jump-robust volatility estimation using nearest neighbor truncation. J Econ.

[CR4] Bai J, Perron P (2003). Critical values for multiple structural change tests. Econ J.

[CR5] Barndorff-Nielsen OE, Shephard N (2004). Power and bipower variation with stochastic volatility and jumps. J Financ Econ.

[CR6] Barunika J, Krehlika T, Vachaa L (2016). Modeling and forecasting exchange rate volatility in time-frequency domain. Eur J Oper Res.

[CR7] Cervelló-Royo R, Guijarro F, Michniuk K (2015). Stock market trading rule based on pattern recognition and technical analysis: forecasting the DJIA index with intraday data. Expert Syst Appl.

[CR8] Charles A, Darne O (2014). Large shocks in the volatility of the Dow Jones industrial average index: 1928–2013. J Bank Finance.

[CR9] Corsi F, Renò R (2012). Discrete-Time volatility forecasting with persistent leverage effect and the link with continuous-time volatility modeling. J Bus Econ Stat.

[CR10] Corsi R, Mittnik S, Pigorsch C, Pigorsch U (2008). The volatility of realized volatility. Econ Rev.

[CR11] Dacorogna M, Ulrich M, Richard O, Oliveier P (2001). Defining efficiency in heterogeneous markets. Quant Finance.

[CR12] Dendramis Y, Kapetanios G, Tzavalis E (2014). Level shifts in stock returns driven by large shocks. J Empir Finance.

[CR13] Dendramis Y, Kapetanios G, Tzavalis E (2015). Shifts in volatility driven by large stock market shocks. J Econ Dyn Control.

[CR14] Dionne G, Pacurar M, Zhou XZ (2015). Liquidity-adjusted Intraday Value at Risk modeling and risk management: an application to data from Deutsche Börse. J Bank Finance.

[CR15] Duonga D, Swanson N (2015). Empirical evidence on the importance of aggregation, asymmetry, and jumps for volatility prediction. J Econ.

[CR16] Ewing BT, Malik F (2016). Volatility spillovers between oil prices and the stock market under structural breaks. Glob Finance J.

[CR17] Hong Y, Li H, Zhao F (2004). Out-of-sample performance of discrete-time spot interest rate models. J Bus Econ Stat.

[CR18] Klose J (2014). Determining structural breaks in central bank reaction functions of the financial crisis. J Econ Asymmetries.

[CR19] Liu SW, Tse YK (2015). Intraday Value-at-Risk: an asymmetric autoregressive conditional duration approach. J Econ.

[CR20] Louzis DP, Xanthopoulos-Sisinis S, Refenes RP (2014). Realized volatility models and alternative Value-at-Risk prediction strategies. Econ Model.

[CR21] Martens M, van Dijk D, de Pooter M (2009). Forecasting S&P 500 volatility: long memory, level shifts, leverage effects, day-of-the week seasonality and macroeconomic announcements. Int J Forecast.

[CR22] McAleer M, Medeiros MC (2008). A multiple regime smooth transition heterogeneous autoregressive model for long memory and asymmetries. J Econ.

[CR23] Muller U, Dacorogna M, Dav R, Olsen R, Pictet O, Ward J (1993) Fractals and intrinsic time—a challenge to econometricians. In: XXXIX-th international AEA conference on real time econometrics, pp 14–15

[CR24] Nelson DB (1991). Conditional heteroskedasticity in asset returns: a new approach. Econometrica.

[CR25] Patton AJ (2011). Volatility forecast comparison using imperfect volatility proxies. J Econ.

[CR26] Patton AJ, Sheppard K (2015). Good volatility, bad volatility: signed jumps and the persistence of volatility. Rev Econ Stat.

